# Osteoporosis-Related Randomized Clinical Trials With Middle-Aged and Older Adults Registered on the International Clinical Trials Registry Platform

**DOI:** 10.3389/fendo.2021.702261

**Published:** 2021-08-31

**Authors:** Fenghua Lai, Ling Pei, Xinwen Chen, Jin Li

**Affiliations:** ^1^Department of Endocrinology, The First Affiliated Hospital, Sun Yat-sen University, Guangzhou, China; ^2^Department of Geriatrics, The First Affiliated Hospital, Sun Yat-sen University, Guangzhou, China

**Keywords:** osteoporosis, aging, randomized clinical trial, registry, publication

## Abstract

**Background:**

A better understanding of the current features of osteoporosis-related randomized clinical trials (RCTs) is important for improving clinical trial designs and promoting the translatability of results into benefits for patients. However, there is a lack of thorough evaluation of osteoporosis-related RCTs in middle-aged and older populations. Therefore, this study aimed to investigate the characteristics of registered RCTs on osteoporosis among middle-aged and older adults on the International Clinical Trials Registry Platform (ICTRP).

**Methods:**

Osteoporosis-related RCTs registered on the ICTRP were searched on December 31, 2020. The main features of eligible RCTs were assessed. We searched PubMed, Google scholar, Medline, and Embase databases for the publication status of completed RCTs.

**Results:**

A total of 537 osteoporosis-related RCTs were identified for analysis. The number of registered RCTs increased rapidly in 2005 (N = 47). Of these, 346 (64.4%) RCTs involved only women and 275 (51.2%) were retrospectively registered. Most RCTs were of open-label design (61.3%). The most common primary purpose of osteoporosis-related RCTs was treatment (72.3%). Intervention investigated was mainly focused on medication (62.8%), followed by lifestyle or education (19.0%), and dietary supplement (10.4%). After trial completion, the results of only 140 (35.5%) RCTs were available on the ICTRP, and the publication rate after trial completion was 30.5%.

**Conclusions:**

RCTs on osteoporosis among middle-aged and older adults were dominated by retrospectively registered and open-label trials. Most trials lacked available results and associated publications. More awareness of prospective registration and blinding design in osteoporosis-related RCTs is needed. Further, publication and dissemination of RCTs results should be promoted.

## Introduction

Osteoporosis is a common systemic skeletal disease characterized by decreased bone density and microarchitectural deterioration of bone tissue, with a consequent increase in bone fragility and susceptibility to fracture (1, 2). Osteoporosis is one of the main threats of aging, and its prevalence among people aged over 50 years is 30% in women and 15% in men (3). It has been estimated that approximately 8.9 million osteoporotic fractures occur each year (4). The economic burden of osteoporosis and osteoporotic fractures are substantial (5).

Osteoporosis is induced by complex interactions between genetic metabolic and environmental factors (6, 7). Over the last 50 years, there have been many advances in osteoporosis management. Osteoporosis is no longer considered an inevitable consequence of aging. However, owing to many potentially high-risk patients are underdiagnosed and undertreated, mortality and substantial long-term loss of independence associated with osteoporosis remain challenges (8, 9). To better address osteoporosis management issue, many clinical trials have been conducted around the world.

Well-designed randomized clinical trials (RCTs) are important for the development of clinical medicine. In 2004, the International Committee of Medical Journal Editors (ICMJE) demanded that clinical trials should be registered prospectively in a public registry to ensure process transparency (10). In 2005, the International Clinical Trials Registry Platform (ICTRP) was established by the World Health Organization (WHO). This platform provide public and healthcare providers with a unified portal of access to information about clinical trials conducted worldwide. By 2020, the ICTRP had developed into a platform that merged data from 18 different primary clinical trial registries, which contained the most comprehensive information about clinical trials performed around the world (11).

Conducting osteoporosis-related RCTs can promote the disease management. Timely and comprehensive understanding of the current features of osteoporosis-related RCTs is important to improve clinical trial designs and identify neglected research areas. However, thorough evaluations of osteoporosis-related RCTs in middle-aged and older populations are lacking to date. Therefore, this study aimed to investigate the characteristics of registered RCTs on osteoporosis among middle-aged and older adults on the ICTRP.

## Materials and Methods

### Search Strategy

The study protocol was developed in advance. On December 31, 2020, we performed a survey through the ICTRP search portal (http://apps.who.int/trialsearch) for relevant clinical trials using the main search terms “osteoporosis” or “osteopenia” or “osteoporotic fracture” or “hip fracture” or “bone loss” or “low bone mineral density”. The primary registries were shown in **Supplementary Material 1**. A dataset of 2,411 registered clinical trials was exported as CSV file. The dataset was transferred into Excel to facilitate further data selection and classification.

### Study Selection and Classification

Clinical trials that met the following criteria were included: (1) RCTs related to osteoporosis; (2) RCTs designed specifically for adults aged 50 years or more. Trials were excluded if they were: (1) Observational trials; (2) interventional trials but non-RCTs; (3) trials including participants under 50 years of age; (4) duplicates.

All included clinical trials were classified by two independent researchers (FL and LP) in duplicate. Any disagreements were resolved by consensus. Patient consent was not required in this study. The Research Ethics Committee of the First Affiliated Hospital, Sun Yat-sen University approved this study.

### Data Extraction

Using a predefined data extraction form, two researchers (FL and LP) independently extracted the following variables: type of registration, enrollment status, start date, results of completed trials, funding source, location, center, planned sample size, age of participants, primary purpose, type of intervention, and study design.

### Publication of Included Trials

After identifying trials that had been completed, two researchers (FL and LP) independently searched for publications of all eligible osteoporosis-related RCTs with “completed status” using a standardized strategy. The “publications” field on the ICTRP was identified and used to search for potentially matching publications. We then searched PubMed, Google scholar, Medline, and Embase databases using registration numbers, brief titles, and investigator names in all the fields. Articles published in online or print journals were included. The search for trial publication status was updated and finalized by December 31, 2020. Publication was confirmed by matching the study characteristics outlined on the ICTRP with the description in the published manuscript. If more than one publication was present, the earliest publication that reported primary outcome results and associated with the registration number was chosen. Study protocols, interim analyses, reviews, commentaries, and other non-relevant publications were excluded. Publication was reconfirmed by a third researcher (JL). Inconsistencies were resolved by consensus.

### Statistical Analysis

The continuous variables were reported as medians with the interquartile range (IQR). The values of categorical variables were presented as numbers and percentages. Categorical variable were compared using chi-square or Fisher’s exact tests. Continuous variables were compared using Mann-Whitney U-tests. The cumulative probability of publication in the time after trial completion was calculated by the Kaplan-Meier analysis. All statistical tests were performed using SPSS version 25.0 (IBM Corp., Armonk, NY, USA), with statistically significant differences identified by a two-sided *P* value < 0.05.

## Results

### Distribution of Osteoporosis-Related Clinical Trials

**Figure 1:**
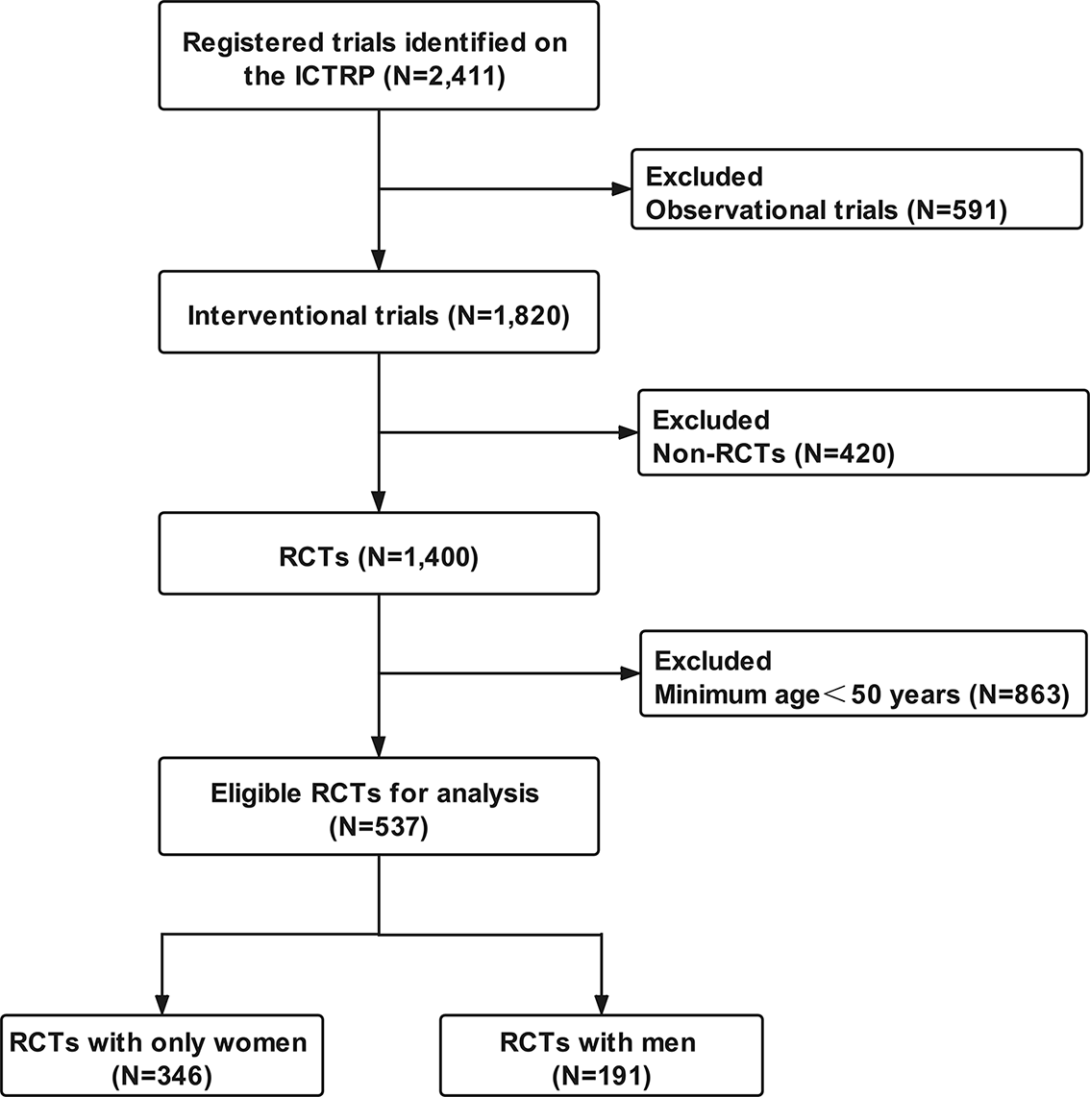
Flow chart of trials selection. ICTRP, International Clinical Trials Registry Platform; RCTs, randomized clinical trials.

**Figure 2:**
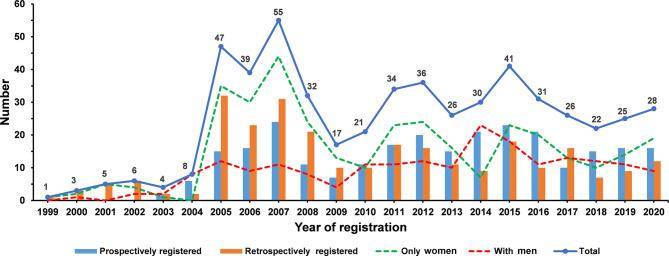
The number of eligible osteoporosis-related randomized clinical trials according to the registered year.

Among the 2,411 osteoporosis-related clinical trials, 591 observational trials and 420 non-RCTs were excluded. After excluding RCTs with participants younger than 50 years (N = 863), 537 RCTs were eligible for analysis. A total of 346 (64.4%) RCTs involved only women and 191 (35.6%) involved men (**Figure 1**). All eligible RCTs were registered during 1999–2020 (**Figure 2**). The annual registered number of osteoporosis-related RCTs increased from 1 in 1999 to a peak of 55 in 2007. The number of registered RCTs increased considerably in 2005 (N = 47). Most RCTs were retrospectively registered between 2005 and 2009. Eligible osteoporosis-related RCTs were identified on the following source registry platforms (**Supplementary Material 2**): ClinicalTrials.gov (298, 55.5%), European Union Clinical Trials Register (72, 13.4%), Japan Primary Registries Network (53, 9.9%), Chinese Clinical Trial Register (39, 7.2%), Australian New Zealand Clinical Trials Registry (24, 4.5%), International Standard Randomized Controlled Trial Number (21, 3.9%), Iranian Registry of Clinical Trials (9, 1.7%), Brazilian Clinical Trials Registry (7, 1.3%), Clinical Trials Registry-India (7, 1.3%), Thai Clinical Trials Registry (3, 0.5%), Clinical Research Information Service, Republic of Korea (2, 0.4%), Pan African Clinical Trial Registry (1, 0.2%), and German Clinical Trials Register (1, 0.2%).

### General Characteristics of Included RCTs

**Table 1** presented a detailed summary of the general characteristics and study design of all eligible RCTs. Regarding type of registration, 51.2% of RCTs were retrospectively registered and 48.8% were prospectively registered. Most RCTs (73.4%) had been completed. However, the results of only 140 (35.5%) RCTs were available on the platform and only 30.5% of RCTs were published. The proportion of RCTs funded by industry was 39.7%. Most RCTs were conducted in Europe (31.3%) and Asia (30.2%), and more than 50% of trials were single-center studies (56.1%). Overall, the planned sample size for recruitment in the RCTs was 160.0 participants. The minimum age of participants for most RCTs (66.3%) was 50–59 years. The most common primary purpose of osteoporosis-related RCTs was treatment (72.3%). Intervention investigated was mainly focused on medication (62.8%), followed by lifestyle or education (19.0%), and dietary supplement (10.4%). More than 60% of RCTs were of open-label design (61.3%). Parallel assignment (89.0%) was the most common intervention model.

**Table 1:** Characteristics and study design of all included RCTs (N = 537).

Item and Subcategory	Number (%) or Median (IQR)
Type of registration	
Prospective	262 (48.8%)
Retrospective	275 (51.2%)
Status	
Completed	394 (73.4%)
Recruiting	62 (11.5%)
Active, not recruiting	35 (6.5%)
Suspended	3 (0.6%)
Terminated	10 (1.9%)
Withdrawn	29 (5.4%)
Unknown status	4 (0.7%)
Results of completed RCTs*^a^*	
No results available	254 (64.5%)
Results available	140 (35.5%)
Publication*^a^*	
Unpublished	274 (69.5%)
Published	120 (30.5%)
Funder	
Industry	213 (39.7%)
Non-industry	324 (60.3%)
Location	
Europe	168 (31.3%)
Asia	162 (30.2%)
North America	120 (22.3%)
Oceania	24 (4.5%)
South America	15 (2.8%)
Africa	2 (0.4%)
Multi-continent	46 (8.5%)
Center	
Single-center	301 (56.1%)
Multi-center	229 (42.6%)
NA	7 (1.3%)
Sample size	160 (74, 400)
Minimum age (years)	
50 to 59	356 (66.3%)
60 to 69	165 (30.7%)
70 or more	16 (3.0%)
Primary purpose	
Treatment	388 (72.3%)
Prevention	118 (22.0%)
Health service	11 (2.0%)
Supportive care	4 (0.7%)
Diagnostic or screening	6 (1.1%)
Basic science	10 (1.9%)
Type of intervention	
Medication	337 (62.8%)
Lifestyle or education	102 (19.0%)
Dietary supplement	56 (10.4%)
Device	13 (2.4%)
Procedure	29 (5.4%)
Masking	
Blinding	208 (38.7%)
Open-label	329 (61.3%)
Intervention model	
Parallel assignment	478 (89.0%)
Sequential assignment	3 (0.6%)
Factorial assignment	12 (2.2%)
Crossover assignment	30 (5.6%)
NA	14 (2.6%)

a The sum of number was the number of completed RCTs.

IQR, inter-quartile range; NA, not available; RCTs, randomized clinical trials.

### Selected Characteristics of Osteoporosis-Related RCTs by Gender

**Figure 3:**
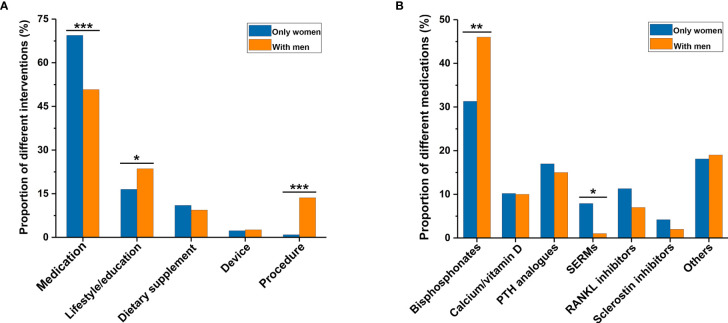
Comparison of interventions between osteoporosis-related randomized clinical trials involving only women and those involving men. **(A)** Type of general intervention between the two groups. **(B)** Type of medication between the two groups. *P < 0.05, **P < 0.01, ***P < 0.001. PTH, parathyroid hormone; SERMs, selective estrogen receptor modulators.

When comparing the characteristics of osteoporosis-related RCTs with only women and those with men (**Table 2**), more RCTs with only women were completed than RCTs with men (78.6% *versus* 63.9%, *P* = 0.004). There were no significant differences in availability of results and publications of completed RCTs between the two groups. More RCTs with only women were funded by industry (48.8% *versus* 23.0%), more frequently conducted in Europe (35.3% *versus* 24.1%), and more often multicenter (47.1% *versus* 34.6%) than RCTs with men (all *P* < 0.001). In terms of the primary purpose of treatment, there were more RCTs with only women than those with men (77.4% *versus* 62.8%, *P* < 0.001). **Figure 3** showed a comparison of interventions between RCTs with only women and those with men. Fewer RCTs with men focused on drugs than RCTs with only women (50.8% *versus* 69.4%, *P* < 0.001). However, more RCTs with men investigated lifestyle or education (23.6% *versus* 16.5%, *P* = 0.045), and procedure (13.6% *versus* 0.9%, *P* < 0.001), than RCTs with only women (**Figure 3A**). Of drug-related RCTs (**Figure 3B**), more RCTs on selective estrogen receptor modulators featured only women compared with men (7.9% *versus* 1.0%, *P* = 0.013), but fewer RCTs on bisphosphonates featured only women compared with men (31.3% *versus* 46.0%, *P* = 0.009). There was no significant difference between RCTs with only women and those with men on parathyroid hormone analogs (17.0% *versus* 15.0%, *P* = 0.649), calcium or vitamin D (10.2% *versus* 10.0%, *P* = 0.958), RANKL inhibitors (11.3% *versus* 7.0%, *P* = 0.223), and sclerostin inhibitors (4.2% *versus* 2.0%, *P* = 0.323).

**Table 2:** Characteristics and study design of RCTs according to the participants.

Variables	Only women (N = 346)	With men (N = 191)	*P*-value
Type of registration			0.077
Prospective	159 (46.0%)	103 (53.9%)	
Retrospective	187 (54.0%)	88 (46.1%)	
Status			0.004
Completed	272 (78.6%)	122 (63.9%)	
Recruiting	30 (8.7%)	32 (16.8%)	
Active, not recruiting	20 (5.8%)	15 (7.9%)	
Suspended	0	3 (1.6%)	
Terminated	5 (1.4%)	5 (2.6%)	
Withdrawn	3 (0.9%)	1 (0.5%)	
Unknown status	16 (4.6%)	13 (6.8%)	
Results of completed RCTs*^a^*			0.094
No results available	168 (61.8%)	86 (70.5%)	
Results available	104 (38.2%)	36 (29.5%)	
Publication*^a^*			0.363
Unpublished	193 (71.0%)	81 (66.4%)	
Published	79 (29.0%)	41 (33.6%)	
Funder			<0.001
Industry	169 (48.8%)	44 (23.0%)	
Non-industry	177 (51.2%)	147 (77.0%)	
Location			<0.001
Europe	122 (35.3%)	46 (24.1%)	
Asia	78 (22.5%)	84 (44.0%)	
North America	75 (21.7%)	45 (23.6%)	
Oceania	14 (4.0%)	10 (5.2%)	
South America	14 (4.0%)	1 (0.5%)	
Africa	1 (0.3%)	1 (0.5%)	
Multi-continent	42 (12.1%)	4 (2.1%)	
Center			<0.001
Single-center	177 (51.2%)	124 (64.9%)	
Multi-center	163 (47.1%)	66 (34.6%)	
NA	6 (1.7%)	1 (0.5%)	
Sample size	160 (68.5, 433)	180 (80, 400)	0.751
Minimum age (years)			0.251
50 to 59	238 (68.8%)	118 (61.8%)	
60 to 69	98 (28.3%)	67 (35.1%)	
70 or more	10 (2.9%)	6 (3.1%)	
Masking			0.095
Blinding	125 (36.1%)	83 (43.5%)	
Open-label	221 (63.9%)	108 (56.5%)	
Intervention model			0.249
Parallel assignment	302 (87.3%)	176 (92.2%)	
Sequential assignment	3 (0.9%)	0	
Factorial assignment	9 (2.6%)	3 (1.6%)	
Crossover assignment	20 (5.8%)	10 (5.2%)	
NA	12 (3.4%)	2 (1.0%)	
Primary purpose			<0.001
Treatment	268 (77.4%)	120 (62.8%)	
Prevention	60 (17.3%)	58 (30.4%)	
Health service	2 (0.6%)	9 (4.7%)	
Supportive care	2 (0.6%)	2 (1.0%)	
Diagnostic or screening	4 (1.2%)	2 (1.0%)	
Basic science	10 (2.9%)	0	

Data were expressed as number (percentage) or median (inter-quartile range).

a The sum of number was the number of completed RCTs.

NA, not available; RCTs, randomized clinical trials.

### Publication of Completed Osteoporosis-Related RCTs

**Figure 4:**
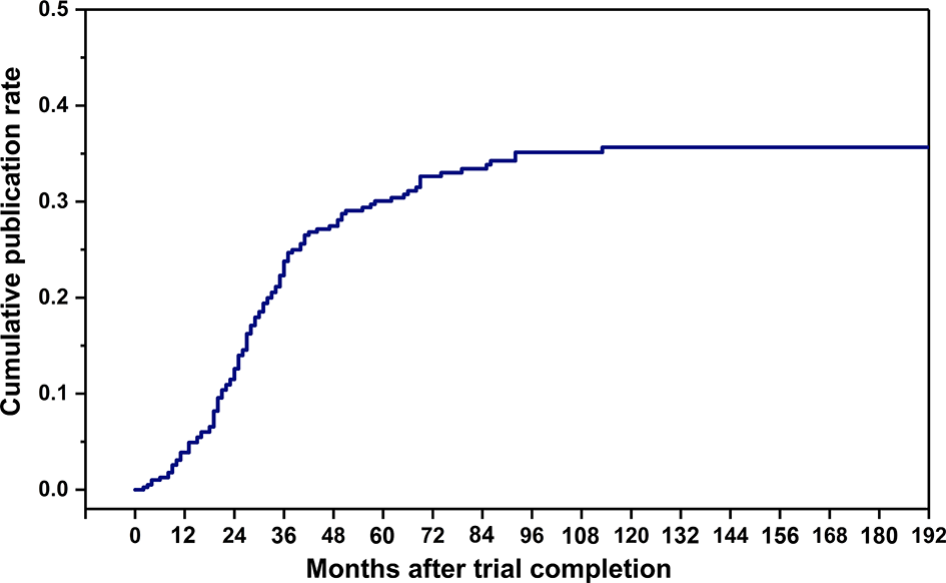
Cumulative publication rate curve after trial completion.

The cumulative publication rate of completed osteoporosis-related RCTs was shown in **Figure 4**. The 1-, 2-, 3-, and 5-year publication rates were 3.9%, 12.6%, 23.8%, and 30.1%, respectively. **Table 3** showed characteristics of the published and unpublished RCTs. More published RCTs than unpublished RCTs were retrospectively registered (70.0% *versus* 50.7%, *P* < 0.001) and had results available on the platform (45.0% *versus* 31.4%, *P* = 0.009). Published RCTs more frequently reported positive outcomes (84.2%). More published RCTs were non-industry funded (61.7% *versus* 50.7%, *P* = 0.045) and more conducted in North America (39.1% *versus* 18.2%, *P* < 0.001) than unpublished RCTs. Published RCTs recruited larger target samples than unpublished RCTs [median, 255.5 (112.5, 1055.0) *versus* 144.5 (60.0, 283.5), *P* < 0.001]. More published RCTs than unpublished RCTs were designed with blinding (50.0% v*versus* 32.1%, *P* = 0.040).

**Table 3:** Characteristics and study design of the completed RCTs according to the publication status.

Variables	Published (N=120)	Unpublished (N=274)	*P*-value
Type of registration			<0.001
Prospective	36 (30.0%)	135 (49.3%)	
Retrospective	84 (70.0%)	139 (50.7%)	
Results of completed trials			0.009
No results available	66 (55.0%)	188 (68.6%)	
Results available	54 (45.0%)	86 (31.4%)	
Outcome			—
Positive	101 (84.2%)	—	
Negative	19 (15.8%)	—	
Funder			0.045
Industry	46 (38.3%)	135 (49.3%)	
Non-industry	74 (61.7%)	139 (50.7%)	
Location			<0.001
Europe	35 (29.2%)	95 (33.7%)	
Asia	14 (11.7%)	91 (33.2%)	
North America	47 (39.1%)	50 (18.2%)	
Oceania	4 (3.3%)	7 (2.6%)	
South America	0	10 (3.6%)	
Africa	0	2 (0.7%)	
Multi-continent	20 (16.7%)	19 (6.9%)	
Center			0.682
Single-center	62 (51.7%)	140 (51.1%)	
Multi-center	56 (46.6%)	132 (48.2%)	
NA	2 (1.7%)	2 (0.7%)	
Participants			0.363
Only women	79 (65.8%)	193 (70.4%)	
With men	41 (34.2%)	81 (29.6%)	
Sample size	255.5 (112.5, 1055.0)	144.5 (60.0, 283.5)	<0.001
Minimum age (years)			0.168
50 to 59	80 (66.7%)	197 (71.9%)	
60 to 69	33 (27.5%)	73 (26.6%)	
70 or more	7 (5.8%)	4 (1.5%)	
Primary purpose			0.076
Treatment	79 (65.8%)	207 (75.5%)	
Prevention	33 (27.5%)	58 (21.2%)	
Health service	3 (2.5%)	2 (0.7%)	
Supportive care	2 (1.7%)	0	
Diagnostic or screening	2 (1.7%)	1 (0.4%)	
Basic science	1 (0.8%)	6 (2.2%)	
Type of intervention			0.073
Medication	71 (59.2%)	188 (68.6%)	
Lifestyle or education	25 (20.8%)	45 (16.4%)	
Dietary supplement	17 (14.2%)	26 (9.5%)	
Device	3 (2.5%)	7 (2.6%)	
Procedure	4 (3.3%)	8 (2.9%)	
Masking			0.040
Blinding	60 (50.0%)	88 (32.1%)	
Open-label	60 (50.0%)	186 (67.9%)	
Intervention model			0.132
Parallel assignment	110 (91.7%)	236 (86.1%)	
Sequential assignment	1 (0.8%)	1 (0.4%)	
Factorial assignment	5 (4.2%)	7 (2.6%)	
Crossover assignment	3 (2.5%)	19 (6.9%)	
NA	1 (0.8%)	11 (4.0%)	

Data were expressed as number (percentage) or median (inter-quartile range).

NA, not available; RCTs, randomized clinical trials.

## Discussion

This is the first study to comprehensively analyze the characteristics of registered RCTs on osteoporosis in middle-aged and older adults. Our results showed that the number of registered osteoporosis-related RCTs increased rapidly in 2005. These RCTs were dominated by retrospectively registered and open-label trials. The osteoporosis-related RCTs mainly focused on women and drug-related treatment. After trial completion, most RCTs had no results available on the platform, and the publication rate was <40%.

The number of osteoporosis-related RCTs registered on the ICTRP markedly increased in 2005 after the implementation of the ICMJE policy that required trials to be registered to be considered for publication, similar to trials focused on other diseases (12). Prospective registration in a public registry is important for improving the transparency and quality of clinical trials (10, 13). However, the present results showed that more than 50% of osteoporosis-related RCTs were retrospectively registered. Previous studies have also shown that approximately half of clinical trials registered on the ICTRP are retrospective (14, 15). Another study showed that among clinical trials published in a group of medical journals in 2013, only 31% were prospectively registered (16). Similarly, the present study found that 70% of published osteoporosis-related RCTs were retrospectively registered. Lack of awareness about clinical trial registration may be a main contributing factor. Investigators were forced to register at the publication stage. A survey on trialist attitudes toward clinical trial registration demonstrated that almost one-third of trialists cited lack of knowledge about trial registration as a key reason for failure to follow the prospective registration policy (17). Awareness of clinical trial prospective registration needs to be improved.

The present findings indicate that more than 60% of osteoporosis-related RCTs were of open-label design. Clinical trials designed without blinding may exaggerate the benefits of intervention by 14% (18). In RCTs, expectations of investigators and participants may generate powerful induction effects. Negative expectations may generate placebo effects, whereas positive expectations may enhance intervention effects (19). To lessen the effect of expectations, a blinding design is often used in RCTs to evaluate the specific effect of a novel intervention. In practice, however, some types of intervention, such as lifestyle or educational interventions, limit the implementation of blinding design (20). In our study, 73.2% of interventions used drug and dietary supplementation, which are appropriate for the implementation of blinding. However, only 38.7% of RCTs were blinding, indicating that there is a need for expansion of blinding design in RCTs.

There was a disparity in the gender of RCT participants in this study. Although osteoporosis is usually considered as a female disease, 1 in 8 men older than 50 years suffer from a fragility fracture (21). Women experience rapid bone loss after menopause (22). Instead, men undergo a slow bone loss with age (23). This slow bone loss had an average rate of 0.5% to 1.0% per year, and eventually increased the incidence of fractures (24). Almost one third of fractures and one-quarter of the total cost burden of osteoporosis were borne by men (25). Despite this substantial disease burden in men, fewer RCTs on the ICTRP focused on osteoporosis in men.

In the past two decades, many drugs for anti-osteoporosis have been introduced, and led to active trials of osteoporosis (26). Our study showed that medication (mainly focusing on bisphosphonates and parathyroid hormone analogs) was the most frequently identified intervention among osteoporosis-related RCTs. Although drug treatments and recommendations for osteoporosis therapy are known effective, many patients at risk of fracture are still underdiagnosed and undertreated (27). The reasons for this are complicated and multifactorial, including healthcare systems, providers, and patient barriers (28). Previous studies indicated that less than 1 in 5 osteoporosis patients receive care or education to prevent future fractures (29, 30). To better address this care gap of osteoporosis, clinical trials regarding health services or prevention are needed, but these types of RCTs comprised only 24% of osteoporosis-related RCTs on the ICTRP.

The systematic reporting and publication of clinical trials results provide a reliable basis for evidence-based medicine and promote the development of clinical medicine and public health (31). In the present study, most osteoporosis-related RCTs had no available results on the ICTRP, and the publication rate of RCTs with completed status was less than 40%. Ross et al. reported that more than 50% of completed trials registered on ClinicalTrials.gov failed to publish (32). Underreporting of clinical trials results may induce biased evidence, with adverse consequences for clinical practice and research (33). Selective publication was a main factor that affected the publication of clinical trial (34). If the trial results contradict the investigators’ beliefs or put sponsors at financial risk, publications may be delayed or suppressed (35). Furthermore, investigators, editors, and reviewers were generally less excited about negative trials but more enthusiastic about positive or equivalent trials.^10^ Similarly, more than 80% of published osteoporosis-related RCTs in the present study reported positive outcomes.

This study had several limitations. First, some clinical trials whose protocols had not been registered on online platforms might be missed. Second, this study only analyzed the general characteristics of registered osteoporosis-related RCTs. The further strengths and weaknesses of the RCTs were difficult to evaluated because of limited information. Finally, all information of clinical trials on the platform were reported by researchers, and we fail to validate of all trial information on the ICTRP. Additionally, not all trials on the platform had up-to-date and complete data.

In conclusion, this study provided useful information that was important for guidance in future clinical trials on osteoporosis treatment and prevention among middle-aged and older adults. Our study showed that osteoporosis-related RCTs were dominated by retrospectively registered and open-label trials. Most RCTs lacked available results and associated publications. There is a need for greater awareness of prospective registration and blinding in osteoporosis-related RCTs. Further, publication and dissemination of clinical trial results should be promoted.

## Data Availability Statement

The original contributions presented in the study are included in the article/**Supplementary Material**. Further inquiries can be directed to the corresponding author.

## Author Contributions

FL and LP – trials search, data collection, data analysis and interpretation, writing the draft manuscript. XC – data analysis and critical revision of the manuscript. JL – study conception, data collection, methodology, and interpretation, critical revision of the manuscript and final approval. All authors contributed to the article and approved the submitted version.

## Conflict of Interest

The authors declare that the research was conducted in the absence of any commercial or financial relationships that could be construed as a potential conflict of interest.

## Publisher’s Note

All claims expressed in this article are solely those of the authors and do not necessarily represent those of their affiliated organizations, or those of the publisher, the editors and the reviewers. Any product that may be evaluated in this article, or claim that may be made by its manufacturer, is not guaranteed or endorsed by the publisher.
