# Supplementary material for: Osteoporosis-Related Randomized Clinical Trials With Middle-Aged and Older Adults Registered on the International Clinical Trials Registry Platform
Source: Front Endocrinol (Lausanne). 2021 Aug 31;12:702261. doi: 10.3389/fendo.2021.702261 (PMC8438405; doi:10.3389/fendo.2021.702261)
Supplement: Supplementary file 1 [file DataSheet_1.pdf]

Supplementary Materials

Supplementary Material 1. Source registries on the ICTRP

Source registry	Country or region	Website	Language
ANZCTR	Australia and New Zealand	http://www.anzctr.org.au/	English
ChiCTR	China	http://www.chictr.org.cn/	Chinese, English
ClinicalTrials.gov	United States	http://www.clinicaltrials.gov/	English
CRiS	Korea	http://cris.nih.go.kr/	Korean, English
CTRI	India	http://www.ctri.nic.in/	English
DRKS	Germany	http://www.germanctr.de/	German, English
EU-CTR	Europe	https://www.clinicaltrialsregister.eu/	English
IRCT	Iran	http://www.irct.ir/	Arabic, English
ISRCTN	United Kingdom	http://www.isrctn.org/	English
JPRN	Japan	http://rctportal.niph.go.jp/	Japanese, English
LBCTR	Lebanon	https://lbctr.moph.gov.lb/	Arabic, English
NTR	Netherlands	http://www.trialregister.nl/	English
PACTR	Africa	http://www.pactr.org/	English
ReBec	Brazil	http://www.ensaiosclinicos.gov.br/	English, Portuguese
REPEC	Peru	https://ensayosclinicos-repec.ins.gob.pe/en/	Spanish, English
RPCEC	Cuba	http://registroclinico.sld.cu/	Spanish, English
SLCTR	Sri Lanka	http://www.slctr.lk/	English
TCTR	Thailand	http://www.clinicaltrials.in.th/	Thai, English

ICTRP, International Clinical Trials Registry Platform; ANZCTR, Australian New Zealand Clinical Trials Registry; ChiCTR, Chinese Clinical Trial Register; CRiS, Clinical Research Information Service, Republic of Korea; CTRI, Clinical Trials Registry-India; DRKS, Deutschen Register Klinischer Studien (German Clinical Trials Register); EU-CTR, European Union Clinical Trials Register; IRCT, Iranian Registry of Clinical Trials; ISRCTN, International Standard Randomized Controlled Trial Number; JPRN, Japan Primary Registries Network; LBCTR, Lebanese Clinical Trials Registry; NTR, The Netherlands National Trial Register; PACTR, Pan African Clinical Trial Registry; ReBeC, Brazilian Clinical Trials Registry; REPEC, Peruvian Clinical Trial Registry; RPCEC, Cuban Public Registry of Clinical Trials; SLCTR, Sri Lanka Clinical Trials Registry; TCTR, Thai Clinical Trials Registry.

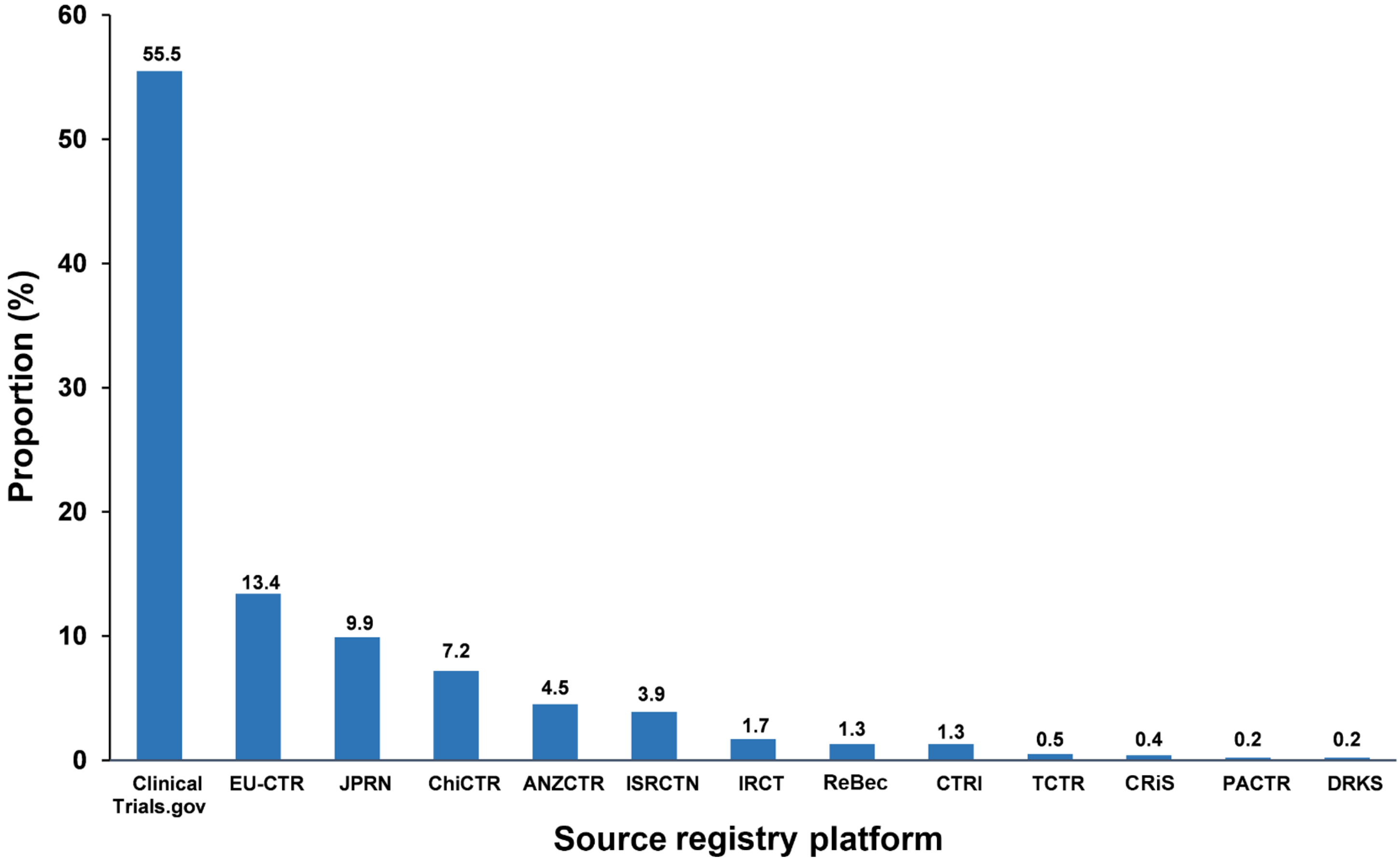

Supplementary Material 2. Source registry platforms of eligible osteoporosis-related randomized clinical trials.
